# Automatic left ventricular analysis with Inline VF performs well compared to manual analysis: results from Barts Cardiovascular Registry

**DOI:** 10.1186/1532-429X-18-S1-P31

**Published:** 2016-01-27

**Authors:** Mihir M Sanghvi, Patricia Feuchter, Filip Zemrak, Redha Boubertakh, Avan Suinesiaputra, Alistair Young, Roshan Weerackody, Neha Sekhri, Anna S Herrey, Charlotte Manisty, Ceri Davies, Mark Westwood, James Moon, Saidi A Mohiddin, Steffen E Petersen

**Affiliations:** 1 Cardiovascular Imaging, Barts Heart Centre, London, UK; 2 grid.4868.20000000121711133Centre of Advanced Cardiovascular Imaging, William Harvey Research Institute, London, UK; 3 grid.9654.e0000000403723343Department of Anatomy with Radiology, University of Auckland, Auckland, New Zealand

## Background

Manual left ventricular (LV) volumes and function analysis is time consuming and operator dependent. Automated and semi-automated LV analysis tools could be helpful, especially in high volume clinical and research centres. Inline VF (Siemens) is a fully-automated assessment tool performing LV volume analysis during scan acquisition.

The aims of this study were: 1) to assess performance of Inline VF against manual analysis of LV volumes and function, 2) to derive conversion formulas from linear regression models and 3) to validate adjusted Inline VF parameters to ascertain whether this improves accuracy of the automated method.

## Methods

In stage one, we included 218 randomly selected participants of Barts Cardiovascular Registry who underwent cardiac magnetic resonance (CMR) imaging with 1.5T Aera or 3T Prisma Siemens scanners for various clinical reasons between 03/12/2014 and 31/07/2015.

Fully automated LV analysis was performed with Inline VF after acquiring a short axis cine stack with retrospective balanced steady state free precession sequence. Inline VF data was extracted from DICOM files with a MATLAB script. Various clinical operators performed manual LV analysis of the same images on cvi^42^ software.

We excluded 25 participants (11.5%) with clearly misplaced Inline VF contours or misrecognised mitral valve on visual assessment and a further two with missing data from manual analysis, leaving 193 studies with full datasets. We used Pearson's correlation coefficients and two-way random single measures agreement intraclass correlation coefficients (ICC) to compare LV end-diastolic volume (EDV), end-systolic volume (ESV), stroke volume (SV) and ejection fraction (EF) from manual and Inline VF methods. The limits of agreement between measurements were also compared using the Bland-Altman plots for visual analysis.

In stage two, we included 77 separate studies. We calculated corrected EDV and ESV by applying the conversion formulas derived in stage one: EDVcorr = 16.8+inlineVF EDV*0.88 and ESVcorr= -3.8+inlineVF ESV*0.87. We then repeated the analysis from stage one.

## Results

There was an excellent agreement (ICC>0.9) between the directly measured volumes: EDV and ESV were 1.9% and 20.9% larger on Inline VF respectively. Parameters calculated from the measured volumes - SV and EF - had good agreement (ICC >0.72), however with larger mean differences and weaker correlation.

The agreement of all parameters improved after correction in Stage 2.

Detailed results are presented in the figures.

## Conclusions

Inline VF automated LV analysis tool performs well compared to manual analysis. Accuracy of ESV, SV and EF measurements improves markedly following mathematical correction. We note exclusion of 11.5% of study participants due to anatomical misrecognition by the software. With further adjustments to the automated algorithm, the tool could find application in high volume research studies, but also in clinical departments in identifying abnormal scans and prioritising analysis/reporting.

**Figure 1:**
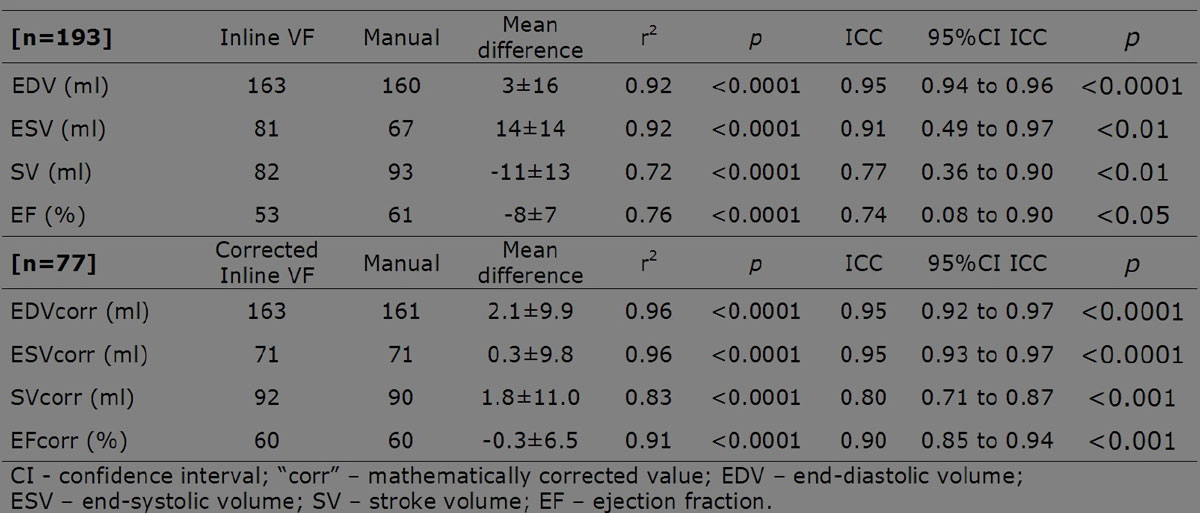
**Comparison of manual, automated (Inline VF) and corrected automated analysis of left ventricular volumes and function with Pearson's correlation and intraclass correlation coeffiecients (ICC)**.

**Figure 2:**
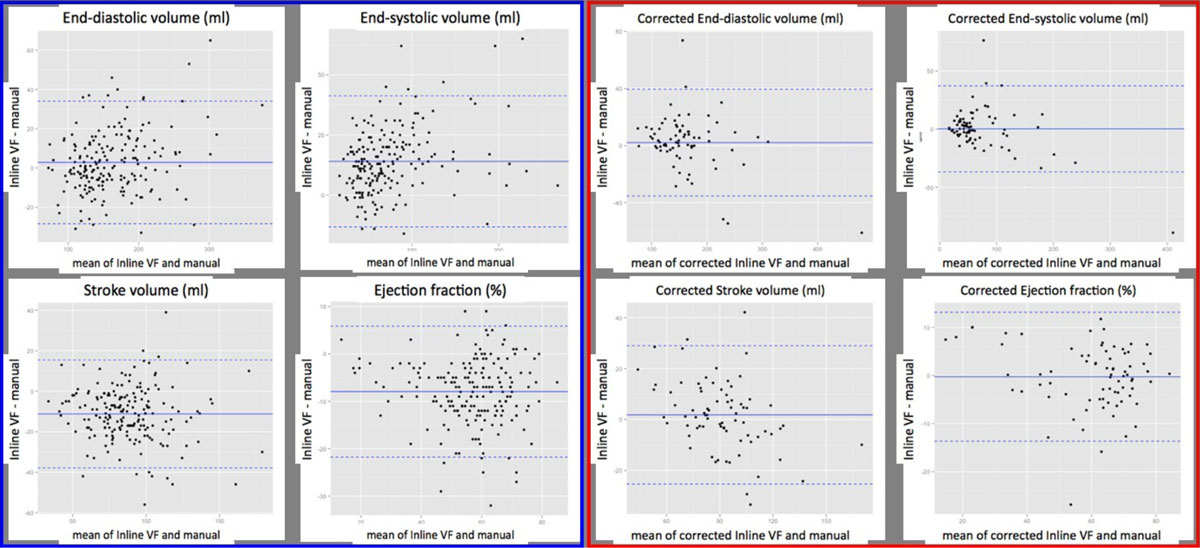
**Bland-Altman plots showing differences between Inline VD (original - blue box, corrected - red box) and manual analysis of LV volumes and ejection fraction.**

